# A Fixed Flow is More Effective than Titrated Flow during Bubble Nasal CPAP for Respiratory Distress in Preterm Neonates

**DOI:** 10.3389/fped.2015.00081

**Published:** 2015-10-12

**Authors:** Srinivas Murki, Ratan Kumar Das, Deepak Sharma, Praveen Kumar

**Affiliations:** ^1^Neonatal Unit, Department of Pediatrics, Fernandez Hospital, Hyderabad, India; ^2^Neonatal Unit, Department of Pediatrics, Advanced Pediatrics Center, Postgraduate Institute of Medical Education and Research, Chandigarh, India

**Keywords:** bubble CPAP, fixed flow, preterm, respiratory distress, titrated flow

## Abstract

**Background:**

The clinical effects of a pre-fixed flow of air-oxygen versus a flow titrated according to visible bubbling are not well understood.

**Objective:**

To compare the effects of a fixed flow (5 L/min) and titrated flow (flow just enough to ensure bubbling) at different set pressures on delivered intra-prong pressure, gas exchange and clinical parameters in preterm infants on bubble CPAP for respiratory distress.

**Methods:**

Preterm infants <35 weeks gestational age on bubble CPAP and <96 h of age were enrolled in this crossover study. They were subjected to 30-min periods of titrated flow and fixed flow. At the end of both epochs, gas flow rate, set pressure, FiO_2_, SpO_2_, Silverman retraction score, respiratory rate, abdominal girth, and blood gases were recorded. The delivered intra-prong pressure was measured by an electronic manometer.

**Results:**

69 recordings were made in 54 infants. For each of the set CPAP pressures (4, 5, and 6 cm H_2_O), the mean delivered pressure with a fixed flow of 5 L/min was higher than that delivered by the titrated flow. During the fixed flow epoch, the delivered pressure was closer to and higher than the set pressure resulting in higher PaO_2_ and lower PaCO_2_ as compared to titrated flow epoch. In the titrated flow period, the delivered pressure was consistently lower than the set pressure.

**Conclusion:**

In preterm infants on bubble CPAP with set pressures of 4–6 cm H_2_O, a fixed flow of 5 L/min is more effective than a flow titrated to ensure adequate visible bubbling. It achieves higher delivered pressures, better oxygenation and ventilation.

## Introduction

Bubble continuous positive airway pressure (B-CPAP) is the standard widely used cost-effective intervention for supporting preterm infants with respiratory distress (1). The pressure in B-CPAP circuit is generated by creating an obstruction to the flow of gases by using a column of water. A sufficient gas flow rate is required to prevent re-breathing of carbon dioxide and to compensate for leaks around connecters and nasal prongs. Usually, a flow rate of 5–10 L/min is recommended for neonates on B-CPAP (2).

Experiments using *in vitro* lung models have shown that increasing bias flow increases the delivered intra-prong pressure in B-CPAP system, even in the presence of appreciable nares-prong leaks (3, 4). Similar observations were confirmed in preterm infants (5). However, the clinical effects of changing flow and its impact on gas exchange are not clearly known. In this crossover study, we compared the effects of a fixed flow (5 L/min) versus titrated flow (flow just adequate to generate bubbling) on delivered intra-prong pressures, improvement in respiratory distress and gas exchange in preterm infants receiving B-CPAP for respiratory distress.

## Patients and Methods

This was an open labeled, crossover study conducted in a Level III neonatal intensive care unit from April 2012 to December 2012. The study protocol was approved by the ethics review board of the institute and a written informed consent was obtained from one of the parents before enrollment.

Preterm infants <35 weeks gestation receiving B-CPAP for respiratory distress and <96 h of life were eligible for the study. Infants with major congenital anomalies and those with metabolic acidosis (pH < 7.20 and base excess more than −12) were excluded. After the infants were enrolled, the CPAP flow was adjusted to generate just adequate bubbling in the water chamber both during inspiration and expiration (titrated flow epoch). Once the infant was stable on B-CPAP (minimal or no retractions, capillary refill time <3 s, SpO_2_ >88% on FiO_2_ <0.40 and heart rate <160/min) for a duration of 30 min, recordings of flow rate, set pressure (depth of water column), fraction of inspired oxygen (FiO_2_), respiratory rate, SpO_2_, Silverman retraction score for severity of respiratory distress (6), abdominal girth, and arterial blood gas were recorded. The intra-prong delivered pressure was monitored with an electronic manometer (differential manometer – Extech Instrumental USA, sensitivity – 0.1 cm, sampling frequency – 5 s) at the pressure port of the Hudson nasal prongs (Hudson RCI^®^). Hudson prongs with the largest size that fitted comfortably in the infants’ nares without blanching the surrounding nasal tissue and disposable bubble CPAP circuits (BC 151, Fisher & Paykel, Auckland, New Zealand) were used for all infants. After obtaining the recordings on titrated flow, the air-oxygen flow was changed to 5 L/min (fixed flow epoch) keeping the set pressure, i.e., immersion depth of water column and FiO_2_) constant. After stabilizing the infant on 5 L/min flow for 30 min, the respiratory rate, SpO_2_, Silverman retraction score, abdominal girth, and delivered intra-prong pressures were measured again. All blood gases were drawn from an indwelling umbilical or peripheral artery catheter. The intra-prong pressures recorded during the last 5 min of the titrated and fixed flow epochs were extracted from the data acquired by the electronic pressure manometer to calculate the average, minimum, and maximum pressures.

Each infant served as its own control. An infant could be included in the study more than once, if the set pressure (immersion depth) had been changed by the treating team based on their clinical judgment and within the first 96 h of life. The existing unit protocol mandated change in CPAP pressure for increasing FiO_2_ requirement (>0.10) associated with low lung volume on chest X ray or increasing respiratory distress (Silverman retraction score) (6). However, each infant was studied only once at a given set pressure (4, 5, or 6 cm).

## Outcomes

The primary study outcome was different between the set ­pressure and average delivered pressure. Other outcomes were noise (maximum–minimum pressure delivered), effect on pH, PaO_2_, PaCO_2_, respiratory rate, Silverman retraction score, and abdominal girth.

## Statistics and Sample Size

Delivered pressure, noise (maximum–minimum delivered pressure), pH, PaO_2_, PaCO_2_, Silverman retraction score (6), and abdominal girth were compared between titrated flow and fixed flow (5 L/min) epochs using paired *t* test. Comparison of differences between set and delivered pressures across the three pre-set CPAP pressures was done by ANOVA. From the downloaded data acquired by the electronic pressure manometer, average, minimum, and maximum delivered pressures were obtained from the last 5 min of the recording in a particular flow epoch.

We assumed that the actual delivered pressure in fixed flow epoch would differ from that delivered during titrated flow epoch by 0.5 cm H_2_O with a SD of 0.3. To be able to detect this with a power of 80% and an alpha error of 0.05, we required 16 observations at each set pressure. Since we used three set pressures of 4, 5, and 6 cm of H_2_O, we required a total of 48 recordings. We included 69 consecutive recordings in the study until we achieved 16 observations at each CPAP set pressure.

## Results

**Figure 1:**
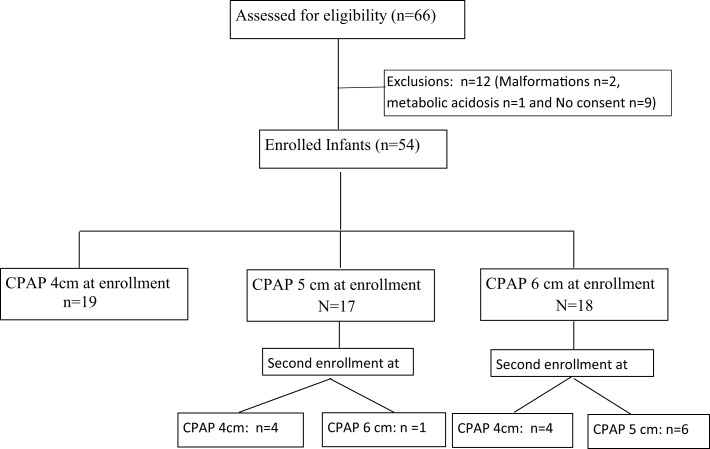
**Flow of patients in the study**.

During the study period, 69 recordings were obtained from 54 infants. Fifteen infants were enrolled twice but at different set CPAP pressures (Figure 1). Among the enrolled infants mean gestational age was 29.9 ± 2.3 weeks (range: 25–34 weeks) and the mean birth weight was 1284 ± 474 g (range: 720–2720 g). The median Silverman score at initiation of CPAP was 5 [interquartile range (IQR) 5–6] and, the median FiO_2_ was 0.25 (IQR 0.21–0.25). The primary cause of respiratory distress was respiratory distress syndrome (*n* = 48) in 70% of the enrolled infants (transient tachypnea of newborn *n* = 17, pneumonia *n* = 4). The median air-oxygen flow during the titrated flow epochs were 3 L/min (IQR 2.5–4 L/min, range: 2–4 L/min), 3 L/min (IQR 2–3 L/min, range: 2–4 L/min), and 3 L/min (IQR 2–3 L/min, range: 2–4 L/min) for set CPAP pressures of 4, 5, and 6 cm, respectively. At all set CPAP pressures from 4 to 6 cm H_2_O, the flow required to generate minimum bubbling was always lower than 5 L/min.

For each of the set CPAP pressure of 4, 5, and 6 cm H_2_O, the delivered pressure with a fixed flow of 5 L/min was higher than that delivered by the titrated flow. The titrated flow achieved pressures lower than the set pressures while the fixed flow achieved pressures closer to and higher than the set pressure (Table 1). During the fixed flow epoch, the difference between set and delivered CPAP pressure (positive change) was maximum at lower set CPAP pressure (0.35, 0.30, and −0.015 cm at 4, 5, and 6 cm H_2_O respectively). On the other hand, during the titrated flow epoch, the difference between set and delivered CPAP pressure (negative change) was maximum at higher set CPAP pressure (−0.64, −0.66, and −0.98 cm at 4, 5, and 6 cm H_2_O, respectively). However the noise (maximum–minimum pressure) delivered at the nasal interface was similar across both arms of the study (Table 2). The median Silverman score and the mean abdominal girth were similar during the two epochs (Table 3). Oxygenation as indicated by median SpO_2_, median PaO_2_, mean OI and ventilation as indicated by median PaCO_2_ were better and mean respiratory rate was lower during fixed flow epoch (Table 3).

**Table 1:** **Delivered pressures at different set CPAP pressures**.

Set pressure	Titrated flow	Fixed flow at 5 L/min	*p*-Value
4 cm H_2_O (*n* = 27)	3.36 (0.31)	4.30 (0.32)	<0.001
5 cm H_2_O (*n* = 23)	4.36 (0.37)	5.32 (0.37)	<0.001
6 cm H_2_O (*n* = 19)	5 (0.37)	6 (0.44)	<0.001

*All pressures in cm H_2_O; all values mean (SD)*.

**Table 2:** **Noise (maximum–minimum delivered pressures) at different set CPAP pressures**.

Set pressure	Titrated flow	Fixed flow at 5 L/min	*p*-Value
4 cm H_2_O (*n* = 27)	0.41 (0.4)	0.5 (0.45)	0.48
5 cm H_2_O (*n* = 23)	0.4 (0.32)	0.37 (0.21)	0.66
6 cm H_2_O (*n* = 19)	0.41 (0.2)	0.41 (0.18)	1

*All pressures in cm H_2_O; all values are mean (SD)*.

**Table 3:** **Comparison of clinical parameters**.

Variable	Titrated flow (*n* **=** 69)	Fixed flow (*n* **=** 69)	*p*-Value
SpO_2_ (%)^a^	94 (92–96)	97 (95–98)	<0.001
Respiratory rate/min^b^	54 (13)	51 (13)	0.002
Silverman score^a^	1 (0–1)	1 (0–1)	0.13
Abdominal girth (cm)^b^	18.7 (3.3)	18.9 (3.4)	0.09
pH^b^	7.3 (0.06)	7.31 (0.07)	0.02
PaO_2_ (mm Hg)^a^	64 (50–76)	68 (50–76)	<0.001
PaCO_2_ (mm Hg)^a^	39 (33–42)	36 (31–41)	0.01
Oxygenation index^b^	2.10 (1.1)	1.88 (0.87)	0.001

*Median (IQR)*.

*Mean (SD)*.

## Discussion

This crossover study compared the effects of titrated versus fixed (5 L/min) gas flow on clinical parameters as well as gas exchange in preterm infants with respiratory distress being managed on bubble CPAP. In the range of 4–6 cm H_2_O of set CPAP pressure, a fixed flow of 5 L/min delivered pressures closer to and higher than the set pressure, whereas, when the flow was subjectively titrated to just achieve bubbling, the delivered pressure at the prongs was significantly lower than the set pressure by as much as 16% or 1 cm. The higher delivered pressure during the fixed-flow epoch explains the reason for better oxygenation (higher SpO_2_ and higher PaO_2_) and better ventilation (lower PaCO_2_ and lower respiratory rate). Also, a better oxygenation index during fixed-flow epoch may be explained by an increase in functional residual capacity as a result of increased delivered pressure. In an experimental lung model, Pillow et al. (3) found that increasing bias flow increased the mean and the magnitude of pressure oscillations at the airway opening and in the lung. In our study, although increased flow increased the delivered pressure, it did not affect the magnitude of pressure oscillations at the prong level. The difference may be related to the leaks, which are inevitable in a real life situation as compared to a lung model. In a randomized crossover trial, Morley et al. (7) evaluated 26 babies treated with Hudson prong bubble CPAP from a bubbling bottle at vigorous, high amplitude (6 L/min) versus slow bubbling (3 L/min) for 30 min. Vigorous versus slow bubbling did not have any effect on the carbon dioxide, oxygenation or respiratory rate, although the pressure delivered was significantly higher at vigorous bubbling. In another trial, Kahn et al. (5) measured intra-prong pressure at three increasing flow settings (flow rate to cause minimal bubbling, +2 L/min, and +4 L/min), repeated for set nasal CPAP of 4 and 6 cm H_2_O in 12 preterm infants. Similar to our results, they concluded that increasing flow rate increased the delivered CPAP pressures. They also found that at a constant flow rate, the difference between set and delivered CPAP pressure is highest at lower set CPAP pressure. They however did not study the effect on oxygenation, carbon dioxide, or work of breathing. Pillow et al. (8) in their study on preterm lambs, reported no differences after 3 h of CPAP with 8 or 12 L/min flow but found better oxygenation and ventilation with bubble CPAP as compared to constant pressure CPAP. This is likely to be because the high fixed flows actually delivered higher pressures as compared to the set pressures, as seen in our study even at 5 L/min.

In our study, we investigated the effects of flow on delivered pressure as well as clinical outcomes in a relatively large number of infants on bubble CPAP. We incorporated all the clinical relevant parameters, such as actual delivered pressures, oxygenation, PaCO_2_, and abdominal girth to evaluate the effects of altering flow. Each infant served as its own control. Apart from the flow changes, all the other parameters, such as circuit, nasal interface, and depth of water column remained constant during both the epochs. The recordings were obtained at the usually used CPAP pressures (4–6 cm H_2_O) in clinical practice. The manometer used was electronic, highly sensitive with a high sampling rate. We, however, did not measure esophageal or pharyngeal pressures, which would have given a closer approximation to actual delivered pressures and did not randomize the order of titrated and fixed flow epochs.

## Conclusion

During bubble CPAP application in preterm neonates the flow is critical to ensure actual delivery of set pressures. A fixed flow fixed of 5 L/min delivers pressures closer to and higher than the set pressures resulting in better oxygenation and ventilation in comparison to subjective titration of flow based on the appearance of bubbling, which delivers lower than set pressures. Hence, in clinical practice, using a fixed flow of 5 L/min would be more effective and simpler to use.

## Conflict of Interest Statement

The authors declare that the research was conducted in the absence of any commercial or financial relationships that could be construed as a potential conflict of interest.
